# From cercariae to chronic inflammation: understanding schistosome infection and host immune responses

**DOI:** 10.3389/fimmu.2025.1729394

**Published:** 2026-01-13

**Authors:** Alessandra Torsello, Caterina Cattani, Christian Napoli, Andrea Cavani, Fernanda Scopelliti

**Affiliations:** 1 PhD School in Traslational Medicine and Oncology, Department of Medical and Surgical Sciences and Translational Medicine, Faculty of Medicine and Psycology, Sapienza University of Rome, Rome, Italy; 2 National Institute for Health, Migration and Poverty (INMP/NIHMP), Rome, Italy

**Keywords:** immune organs, immunity, immunology, immunomodulation, schistosoma, schistosomiasis

## Abstract

Schistosomiasis is a parasitic disease caused by trematodes of the genus Schistosoma, typically found in tropical and subtropical freshwater environments. Recognized by the World Health Organization as a major emerging disease, schistosomiasis is characterized by the parasite’s ability to modulate and evade the host immune system, enabling long-term persistence within the human body. This immunomodulation not only supports chronic infection but also drives disease pathology, particularly through granulomatous inflammation surrounding parasite eggs trapped in host tissues. A deeper understanding of the immunological interactions between Schistosoma spp. and the human host is crucial for guiding the development of novel therapies. This review aims to delineate the immunological dynamics of Schistosoma infection across different stages of disease progression, with a particular focus on site-specific host–parasite interactions that shape both the acute and chronic phases of schistosomiasis.

## Introduction

Schistosomiasis is a parasitic disease caused by trematodes of the genus Schistosoma, typically found in tropical and subtropical freshwater environments (1, 2). Recognized by the World Health Organization as one of the major emerging diseases (3), schistosomiasis poses a significant public health challenge, particularly in low- and middle-income countries (4). Its prevalence is closely linked to poor sanitation, limited access to clean water, and overcrowded living conditions (5–9)—factors that facilitate transmission and hinder effective disease control. The infection is endemic in regions with insufficient healthcare infrastructure (10–13), making coordinated prevention and treatment efforts difficult. Currently, the only available treatment is praziquantel, which is effective at the individual level but insufficient for achieving environmental eradication. Moreover, concerns over the potential emergence of drug-resistant strains of Schistosoma (14–16) have intensified the search for alternative strategies, including vaccines and immunomodulatory therapies (17, 18).

**Figure 1:**
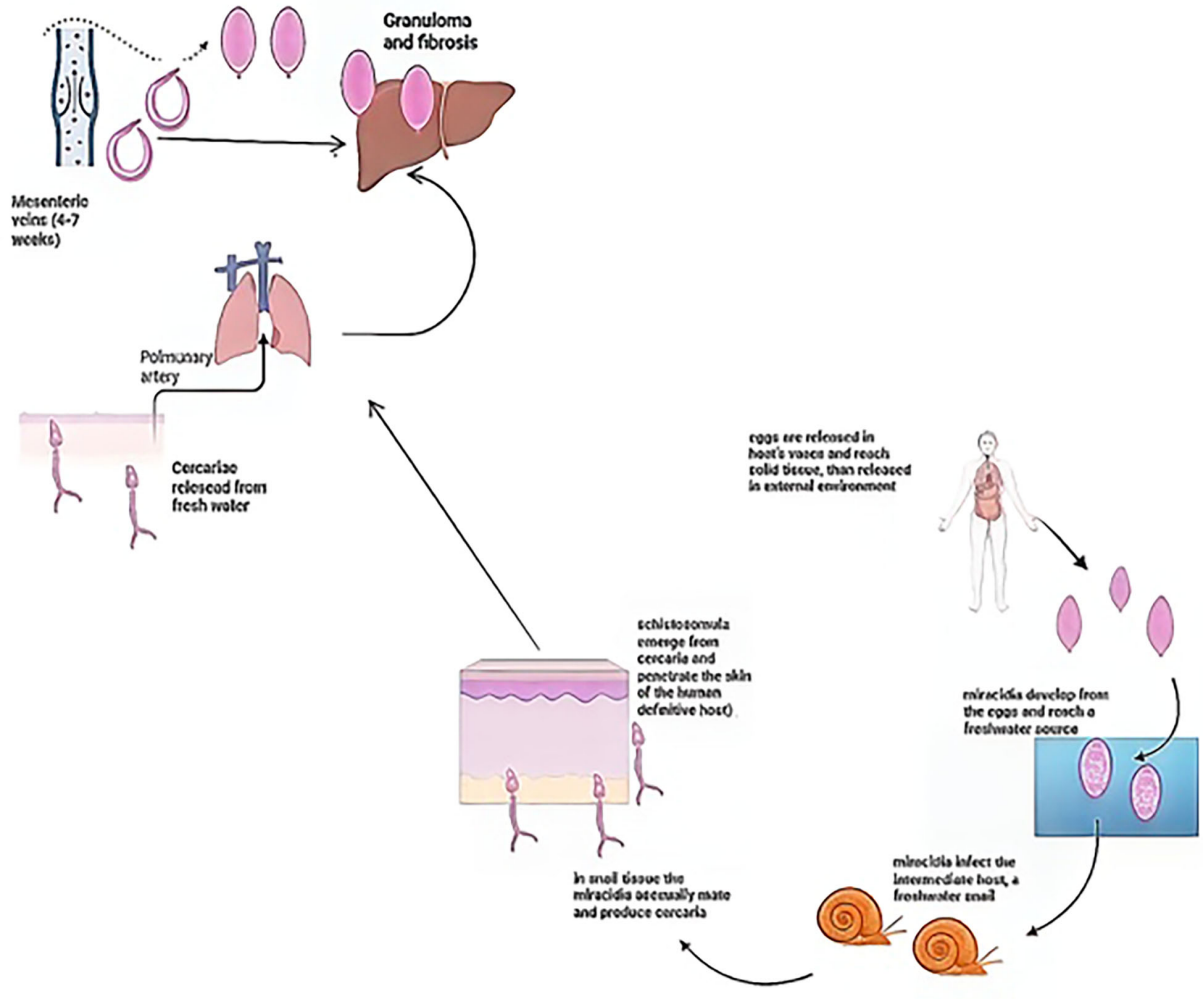
*Schistosoma* life cycle. Schematic representation of the *Schistosoma* life cycle. Eggs released by adult worms in the mesenteric veins are excreted into the external environment and hatch in freshwater, releasing miracidia that infect freshwater snails, the intermediate host. Within the snail, miracidia undergo asexual replication and produce cercariae, which are released into freshwater. Cercariae penetrate human skin, transform into schistosomula, migrate via the lungs, and mature into adult worms in the mesenteric veins. Egg deposition in host tissues induces granuloma formation and fibrosis, particularly in the liver.

Several Schistosoma species can infect humans, but the most clinically relevant are S. japonicum, S. mansoni, and S. haematobium, responsible for intestinal and urinary schistosomiasis (2, 19). The parasite has a complex life cycle involving multiple morphologically distinct stages—miracidia, sporocysts, cercariae, schistosomula, and adult worms—across both intermediate (snail) and definitive (human) hosts (20). Adult worms live and reproduce in the host’s circulatory system, while their eggs migrate through tissues and are excreted to continue the cycle (**Figure 1**) (1, 19).

A defining feature of Schistosoma infection is the parasite’s ability to evade and modulate the host immune response, allowing it to persist in the body for years. Although the host mounts strong immune reactions—especially against migrating larvae and egg antigens—Schistosoma has evolved sophisticated mechanisms to suppress or redirect these responses. This immunomodulation not only facilitates chronic infection but also drives disease pathology, particularly through granulomatous inflammation around eggs trapped in tissues. Understanding the immunological interplay between Schistosoma spp. and the human host is crucial for identifying individuals at risk of severe disease and for guiding the development of novel therapies. Key immune actors involved include macrophages, eosinophils, dendritic cells, regulatory T cells, and T-helper lymphocytes, particularly Th2-skewed responses. These elements shape both innate and adaptive immunity during infection and are targets of parasite-driven immune modulation.

Notably, the immunoregulatory environment induced by schistosomiasis can affect the course of other conditions, including autoimmune and allergic diseases, whose severity and progression may be altered in infected individuals (21). The objective of this review is to delineate the immunological dynamics between Schistosoma spp. and the human host across different stages of infection, with particular focus on the host–parasite interactions in different sites that define both the acute and chronic phases of schistosomiasis.

## Overview of schistosoma infection and migration

Following their release from the snail intermediate host, Schistosoma cercariae penetrate the mammalian host’s skin, initiating the transition from a free-living to a parasitic life stage. Upon entry, they rapidly shed their tails and glycocalyx and differentiate into schistosomula (22), releasing excretory/secretory (E/S) products that facilitate immune evasion by modulating local cutaneous responses. After a short period of residence in the skin, during which they evade both innate and adaptive immune mechanisms, schistosomula enter the dermal vasculature and migrate via the bloodstream to the lungs (23). Despite prolonged exposure to pulmonary immune surveillance, they largely avoid elimination, aided by dynamic changes in tegumental composition, altered glycosylation patterns, and the release of immunomodulatory vesicles. From the lungs, the parasites travel to the liver, where they mature into sexually dimorphic adult worms. These adults establish themselves in the portal or mesenteric venous systems, depending on the species, and begin producing eggs (24). While some eggs are successfully excreted to sustain the transmission cycle, a substantial proportion become trapped in host tissues (25, 26). There, they elicit granulomatous inflammation, which contributes to both the immunopathology and long-term persistence of chronic schistosomiasis (27).

## Cercariae: skin invasion and early immune modulation

Upon contact with a mammalian host, Schistosoma penetrate the skin—a major immunological barrier. The systemic interplay between Schistosoma and host’s immune system begins at this stage. It takes about two hours for the cercariae to lose its tail and remodel its surface membrane and metabolism to adapt to the skin environment; following initial invasion, schistosomula reside in the skin for several days during which several E/S molecules rich in proteases and glycoconjugates are released by the parasite (28, 29). Cercarial proteases exhibit broad substrate specificity against host dermal components (30, 31), and their inhibition significantly compromises the ability of Schistosoma to penetrate and establish within host tissues (32).

The human complement system can recognize and bind to the glycocalyx of Schistosoma cercariae. However, this initial immune response fails to eliminate the parasite or prevent its development into schistosomula, as the cercariae rapidly shed their glycocalyx, thereby discarding bound complement components and evading immune attack (33). Indeed, Wang et al. found that, at least *in vitro*, the complement attack may actually facilitate the metamorphosis of S. japonicum cercariae (34).

Schistosomula secrete molecules that suppress pruritus by acting on MrgprA3^+^ sensory neurons. In the absence of MrgprA3 activation, cutaneous myeloid antigen-presenting cells (APCs) predominantly produce IL-33 rather than IL-17, thereby promoting pathways associated with epidermal hyperplasia and altered skin homeostasis instead of IL-17–driven γδ T-cell recruitment and proliferation (35, 36).

Several studies have highlighted that the immune modulation exerted by cercariae and schistosomula is multifactorial, involving various types of host immune cells and multiple pathways. Notably, these studies underscore the crucial role played by the immunomodulatory cytokine IL-10. IL-10 plays a central role in limiting excessive inflammation by suppressing the activity of pro-inflammatory cytokines such as IFN-γ, TNF-α, and IL-12, and by inhibiting antigen-presenting cell function (37).

Stimulation of human whole-blood cultures with cercarial E/S material caused the early (within 24 h) release of greater quantities of regulatory IL-10 (38). A direct immunomodulatory role of cercariae is supported by experimental evidence showing that radiation-attenuated cercariae fail to induce IL-10 production and, more broadly, a regulatory T cell profile, in contrast to non-attenuated cercariae (39).

Findings from various studies indicate that IL-10 production may occur through distinct immunomodulatory mechanisms activated by Schistosoma. Ramaswamy et al. demonstrated how the parasite secretory products contain a factor that can potentially induce prostaglandin E2 (PGE2) production, that in turn promotes IL-10 secretion from human keratinocytes, although the molecular mechanism of this PGE2 induction from host cells is not fully understood (40). PGE2 may play a dual role: not only by promoting IL-10 production, but also as a potent vasodilator, potentially facilitating Schistosoma penetration into blood vessels.

CD4^+^ T cells became anergic and hyporesponsive following repeated percutaneous injections of Schistosoma larvae in a murine schistosomiasis model, through an IL-10–dependent mechanism (41); Sanin et al. reported similar findings: in mice repeatedly exposed to Schistosoma, an early IL-10–producing CD4^+^ T-cell population suppressed further CD4^+^ T-cell proliferation and the overall immune response in the skin (42). Schistosomula can also directly induce lymphocyte apoptosis: schistosomula secrete antigens capable of upregulating Fas ligand (FasL) expression on lymphocytes, thereby promoting the apoptosis of cutaneous T cells (43).

During the cutaneous phase of Schistosoma infection, not only lymphocyte functions are adversely impaired. Hervé et al. demonstrated that prostaglandin D2 (PGD2), produced by Schistosoma mansoni, inhibits the migration of epidermal Langerhans cells (LCs) to the draining lymph nodes (DLNs) through DP1 receptor stimulation (44). Furthermore, experiments by Angeli et al. suggest that this impaired LC migration may serve as an additional strategy employed by schistosomes to delay and evade the host immune response, with PGD2 playing a pivotal role in modulating cutaneous immune activity (45).

One possible strategy employed by Schistosoma mansoni cercariae may be to leverage host APCs as vehicles for the internalization of their E/S products.

Several proteins contained within the E/S products of Schistosoma mansoni have been shown to bind to mannose receptors (MR) expressed on macrophages. Experimental evidence indicates that macrophages lacking these receptors exhibit a Th1-biased response to the parasite, confirming that MR-mediated uptake of E/S proteins plays a critical role in shaping host immunity (46).

Among the E/S products released by Schistosoma mansoni cercariae were identified proteins belonging to the helminth defence molecule (HDM) family. One of these, Sm16, is internalized by macrophages and shown to modulate their activity and metabolism, leading to a reduced inflammatory response (47).

Sanin et al. demonstrated that Sm16 constitutes a quantitatively relevant fraction of Schistosoma mansoni cercarial E/S products released into the skin during invasion. This protein was shown to impair antigen processing and presentation by macrophages, most likely through interference with endosomal trafficking. These findings highlight Sm16 as a contributor to the immunoregulatory properties of cercarial E/S products and suggest its role in dampening dermal inflammation following percutaneous infection (48).

Another immunomodulatory mechanism employed by Schistosoma cercariae may involve a subset of dendritic cells (DCs) engaged in homeostasis and self-recognition. These DCs interact with authentic cercarial glycosphingolipids containing LeX and pseudo-LeY motifs via the C-type lectin receptor DC-SIGN (49). Additionally, experiments by Jenkins and Mountford support a pivotal role for DCs in promoting a Th2-polarized immune response following interaction with Schistosoma cercariae (50).

Cook et al. demonstrated that eosinophils also contribute to the modulation of the immune response against Schistosoma. In a model of repeated cercarial challenge, they observed a marked eosinophilic infiltration at the site of infection, which created an environment enriched in IL-4 and IL-13 (51). This cytokine milieu promoted the expansion of additional cell populations that suppress the anti-schistosomal immune response, including activated macrophage-like cells expressing arginase-1 and Ym-1, as well as a subset of functionally impaired MHC-II cells. Furthermore, their findings underscore that the development of these suppressive dermal cells is dependent on IL-4Rα signalling. Eosinophils and IL-4 are typically associated with a Th2-type immune response. This provides additional evidence that Schistosoma infection tends to induce a Th2-biased immunological profile from the earliest stages of infection. Studies on cercarial dermatitis further support the emergence of an IL-4–rich environment after cercarial challenge. Cercarial dermatitis, which occurs following repeated Schistosoma infections (52), is characterized by marked eosinophilia and elevated circulating IL-4 levels, highlighting the strong Th2 polarization of the local immune response (53).

## Schistosomula: morphological transformation and pulmonary migration

After shedding its tail and transforming to a schistosomula, the parasite penetrates dermal blood vessels end with the blood flow reaches the lungs. Although species and strains vary in their rate of migration, even the most rapidly migrating species, such as S. japonicum, requires at least five days to reach the bloodstream and subsequently the lungs. That time frame would be expected to allow for the development of a more robust immune response against cercariae or schistosomula compared to that observed in the skin. However, the immune reaction in the lungs appears to resemble the response to a novel antigen, suggesting limited immunological priming during the initial stages of infection (54). Thus despite the exposure of Schistosoma membrane antigens, the host’s immune response remains relatively moderate during the early stages of infection.

Both molecular, *in vitro*, and *in vivo* studies suggest that Schistosoma spp. employs several strategies to evade the immune response throughout its juvenile phase, enabling it to reach full adult form in its final destination within the host’s liver. Attempts to target the migratory phase of Schistosoma infection generally result in disruption of the parasite’s tegument rather than acting against specific protein targets (55, 56). Vaccine prototypes directed at abundant yet less variable tegumental proteins have largely failed, suggesting these proteins are either poorly accessible to the immune system or not critical for parasite survival. In contrast, the most immunogenic schistosomula-derived molecules are the excretory-secretory (ES) products, a complex mixture of proteins and enzymes released primarily from the acetabular glands into host tissues and bloodstream, as well as directly from the tegument (57, 58). These E/S antigens elicit a Th1-skewed immune response (59, 60), unlike the predominantly Th2 responses induced by adult worms or egg antigens (61–63). Nevertheless, also immunization with E/S components has shown limited efficacy in preventing infection or progression to granulomatous pathology (64).

The schistosomula tegument proteome is highly dynamic during development, with many immunogenic apical membrane proteins downregulated compared to cercariae (65). Likewise, the profile of lytic enzymes involved in tissue invasion and immune evasion differs markedly from that of cercariae (65). Glycans on the schistosomula tegument act as pathogen-associated molecular patterns (PAMPs) and are recognized by macrophages and other antigen-presenting cells via TLR. However, the glycosylation pattern of lung-stage schistosomula differs significantly from cercarial and adult stages, with subtle modifications in sugar residues altering antibody binding affinity (66, 67). This variation likely contributes to the ineffectiveness of humoral responses generated during skin invasion (68). Correspondingly, lung schistosomula elicit an innate inflammatory response characterized by innate immune cell recruitment, including γδ T cells (54, 69). Furthermore, the glycosylation state of lipids in schistosomula ES vesicles also differs from adults (70, 71).

Collectively, these data support the hypothesis that schistosomula evade robust immune recognition by continually modifying or masking their most antigenic molecules during this vulnerable developmental window. This antigenic plasticity may also underlie the limited efficacy of vaccines based on schistosomula tegumental antigens in controlling adult worm burden and egg deposition (72).

In addition to antigenic modulation, lung schistosomula actively modulate host cytokine production to attenuate inflammatory damage. Elevated IL-6 levels observed during pulmonary schistosomiasis correlate with a less intense Th1 response and reduced tissue injury, as demonstrated by the heightened inflammation and damage seen in IL-6–deficient mice (73). Moreover, schistosomula tegument preparations induce IL-10 production and recruit regulatory T cells (Tregs) in murine models of airway inflammation, attenuating pro-inflammatory cytokine production and immune activation (74, 75).

The lipid layers at the host-parasite interface also appear critical for schistosomula survival. While their precise immunomodulatory roles remain unclear, disruption of cholesterol in the lipid membrane increases antibody accessibility to membrane markers in S. mansoni but not S. haematobium (76). Alterations to the lipid bilayer can enhance antigen exposure, and schistosomula rely heavily on both active and passive transport through the tegument for nutrient uptake, as they are not yet hematophagous. Nutrient passage can occur even with intact lipid barriers (77), indicating that changes in lipid composition, turnover, or integrity can significantly affect parasite physiology and host interactions. Disruption of sphingomyelin homeostasis, one of the most abundant unsaturated fatty acids in schistosomula lipid bilayers, has been shown to impair parasite growth and development (55). Similarly, treatment of early liver-stage schistosomula with tegument-damaging compounds reduces viability (78).

Complement activation represents a major early immune challenge for schistosomula, which are rapidly targeted predominantly via the alternative pathway. However, after approximately 24 hours, schistosomula evade complement-mediated killing by recruiting host regulatory proteins such as factor H (79). This complement resistance likely contributes to the limited efficacy of many vaccine candidates against cercarial challenge, with some studies showing that complement does not significantly participate in vaccine-mediated parasite killing (80).

## Adult worms: systemic dissemination and long-term immune modulation

Schistosomula migrate through the bloodstream to the portal venous system, where they mature into sexually differentiated adult worms. Depending on the species, worms subsequently relocate to their final niche within either the mesenteric venous system (S. mansoni, S. japonicum) or the venous plexus of the genitourinary tract (S. haematobium) (1, 2). In contrast to cercariae and schistosomula, that evade the host immune system by quickly migrating and constantly changing their surface antigens, adult worms reside stably within specific blood vessels for extended periods and employ distinct long-term immune evasion strategies to persist without eliciting an effective immune response.

As with schistosomula, a key factor is likely the protection conferred at the host–parasite interface by the layers of the tegument. While schistosomula possess a three-layered tegument, adult worms develop a more complex structure composed of seven layers. The outermost layer of adult worm is particularly rich in sphingomyelin, which, according to Migliardo et al., can form a network of hydrogen bonds surrounding the parasite (81). This molecular barrier may shield protein and other immunogenic components of the tegument from recognition by the host immune system. Further studies from the same research group confirmed how adult worms show weaker interaction between outmost layer and the surrounding medium, corroborating the hypothesis that host parasite interface of adult worms is highly masked (82).

Another strategy employed by Schistosoma during its various developmental stages involves the expression of phosphatases on the tegument surface. These enzymes are capable of cleaving host circulating proteins, thereby preventing the activation of the complement system, the coagulation cascade, and other immune responses. The presence of phosphatases and/or suitable protease inhibitors has indeed been demonstrated (83–85). Studies conducted on the serum of infected patients confirm the activation of the complement system and suggest that Schistosoma possesses the ability to degrade activated components, ultimately rendering this immune response ineffective (33). Inal and Schifferli have shown that certain tegumental proteins of Schistosoma can bind to complement components C2 and C4, thereby interfering with the complement cascade (86).

The outermost layers and, more broadly, the tegument of adult worms contain numerous components capable of eliciting a significant Th1 response. Nevertheless, none of the investigated candidates has yet demonstrated sufficient efficacy in preventing cercarial challenge or in inducing effective immunization against adult worms or eggs, largely due to Schistosoma’s sophisticated immunomodulatory strategies.

Several attempts have been made using SWAP (Soluble Worm Antigen Preparation), a mixture of soluble antigens derived from centrifuged adult worms. However, this preparation is largely composed of antigens that are not abundant in the tegument. Proteomic analyses have shown that approximately half of the total SWAP mass consists of cytosolic and cytoskeletal proteins, which are not accessible or exposed to the host immune system. In contrast, only a small fraction—around 3%—corresponds to proteins associated with host-parasite interfaces such as the tegument and gut, which are considered more promising targets for a protective immune response (87).

The sequestration of antigens in regions that are poorly accessible to the host immune system appears to be one of the strategies employed by adult Schistosoma worms. This is further supported by evidence suggesting that significant modifications at the host-parasite interface may explain why certain components of the outer tegument of adult worms fail to elicit the same immune response they are capable of triggering during the lung-stage schistosomula (88). Immunization with antigens derived from adult worms has consistently shown lower protective efficacy compared to those obtained from earlier developmental stages (89), indicating that antigens from adult worms are less accessible to immune recognition. Supporting this, studies in which the tegument is altered or damaged—either directly or through stress conditions—demonstrate that surface antigens become more exposed, resulting in a more robust host immune response (90). In general, compromising the integrity of the tegument is considered an effective strategy for killing adult worms and enhancing the serological response to both adult worm and tegumental antigens, as observed with praziquantel treatment (91).

Some of the most immunogenic proteins may also exhibit significant polymorphism, as exemplified by SjTSP-2. This tegumental protein is highly expressed in adult worms and strongly induces IgG1 and IgG3 responses, yet it is characterized by a high degree of antigenic variability (92). Moreover, SjTSP-2 is not expressed in the tegument during the cercarial stage, but rather in the gut (93), which may explain its limited effectiveness in protecting against cercarial challenge.

Several surface antigens also exhibit significant differences in expression between male and female adult worms (94). As a result, despite the induction of a robust humoral response, infection is not eradicated. More broadly, the proteomic profile of adult worms has been shown to differ between sexes (95, 96), as does the composition and ultrastructure of the tegument (97). These factors may contribute to the distinct immunogenic potential of a given antigen depending on the sex of the parasite.

Schistosoma may not even require broad-spectrum strategies to neutralize the host’s antibody response, as the humoral response is largely focused on a limited set of parasite antigen. Krautz-Peterson et al. have shown that the host’s antibody response to Schistosoma is predominantly directed against a small set of conformational epitopes found on five major tegumental surface membrane proteins. These epitopes appear to dominate the humoral response. Despite the robustness of this circulating anti-tegumental antibody response, neither schistosomula nor adult worms seem to be significantly affected, suggesting that the parasite is able to withstand or evade the immune pressure exerted by these antibodies (98).

All these findings may help explain why, *in vitro*, several components derived from the outermost layer of the adult worm’s tegument have been shown to elicit a canonical Th1 immune response (99), but passive immunization directed versus the same component fail to confer a good protection against the infection (100–103), both in human and animal models or *in vitro* (104).

Schistosoma appears to possess highly effective mechanisms to counteract the host’s IgG-mediated immune response. In contrast, evidence from studies on naturally acquired immunity in resistant individuals suggests that IgE-mediated responses may play a more protective role. This type of immunity seems to develop gradually through repeated exposure to antigens that are normally hidden and only become accessible upon the death of adult worms, which can persist in the human host for several years. Over time, this intermittent antigen exposure promotes the expansion of IgE responses targeting specific schistosome proteins, including members of the tegument-allergen-like (TAL) family. Some of these antigens are shared between adult worms and schistosomula, potentially enhancing cross-stage immune recognition.

The delayed onset of an effective IgE response—often requiring years of infection and multiple reinfection cycles—may be partly explained by the parasite’s ability to interfere with IgE function. For instance, proteases present in the schistosome tegument have been shown to cleave CD23, releasing a soluble fragment that can bind to IgE and inhibits its activity (105).

Further studies have demonstrated that increases in IgE specific to soluble worm antigens (SWA) and Schistosoma protein Sm22.6 correlate positively with pre-treatment Th2 cytokine levels, particularly IL-5, but not with IFN-γ. These associations remain significant even after adjusting for variables such as infection intensity, age, and baseline IgE levels, suggesting that Th2 responsiveness is a key factor in the development of protective IgE responses. Notably, younger children often fail to mount sufficient IgE responses following antigen stimulation, likely due to an underdeveloped Th2 cytokine profile (106).

Genetic studies have also linked resistance to reinfection with specific HLA polymorphisms associated with enhanced IgE production against SWA antigens (107). Moreover, a Th2-skewed immune profile has been shown to confer greater protection in reinfection scenarios (108, 109), while a robust eosinophilic response may further support and modulate Th2 activity (110).

Interestingly, chronic schistosomiasis is often accompanied by an improvement in allergic and atopic conditions, suggesting that the suppression of effective IgE responses may be actively regulated. IL-10 appears to be a central mediator in this process (41, 111, 112), with elevated levels observed during Th2 responses in chronic infection (113). However, the precise mechanisms underlying this immunomodulation remain unclear (114).

Evidence supporting a direct role of adult worm components in inducing IL-10 production remains limited. In human models of single-sex Schistosoma infection—where individuals are exposed exclusively to male or female cercariae—a mixed Th1/Th2 immune response has been observed (115), including in male-only infections (116), without notable IL-10 induction. Similarly, several vaccine trials have elicited a balanced Th1/Th2 response, rather than a regulatory IL-10–dominated profile (100).

In contrast, animal models suggest that female worms may contribute to immune anergy and increased expression of immunoregulatory molecules such as CTLA-4 and IL-10 (117–119), a finding also supported in human studies (115), although in the latter case it is suspected that females may produce unfertilized eggs, potentially influencing the immune profile. Proteomic analyses have revealed sex-specific differences in protein expression (96), including in tegumental proteins (94), suggesting that male and female worms may possess distinct immunomodulatory capacities.

Schistosoma also appears to exert modulatory effects on DCs, the circulating antigen-presenting cells that typically initiate immune responses. In chronic schistosomiasis, both dermal and plasmacytoid DCs have been reported to exhibit an anergic phenotype and reduced expression of Toll-like receptor 2 (TLR2), although the underlying mechanisms remain unclear (120). Similarly, van der Biggelaar et al. observed that DCs in chronically infected individuals display hyporesponsiveness to adult worm antigens (121).

Additional evidence suggests that specific schistosome-derived molecules may actively shape DC function. For instance, lysophosphatidylserine (lyso-PS) from Schistosoma has been shown to induce IL-10–producing regulatory T cells via TLR2-dependent activation of DCs (122). Moreover, the adult worm secretome includes a homolog of human cyclophilin A, which has been demonstrated to act directly on DCs, preferentially promoting the expansion of regulatory T cells (123).

A cysteine protease inhibitor from Schistosoma japonicum (rSj-C) has been shown to impair antigen presentation by DCs. It inhibits lysosomal proteases, reducing MHC class II expression and potentially limiting the ability of DCs to activate adaptive immune responses (124).

Lipids derived from adult Schistosoma mansoni worms have been shown to exert immunomodulatory activity on macrophages, promoting their polarization toward an M2 phenotype (125). The release of metabokines such as succinate and lactate by adult worms, as demonstrated in the study by Skelly and Da’dara, also drives macrophage polarization toward an M2 phenotype (126).

## Eggs: granuloma formation and Th2−biased immune response

Mature parasite pair and migrate to specific vascular niches depending on the species. Schistosoma mansoni and Schistosoma japonicum typically inhabit the mesenteric veins draining the intestines, while Schistosoma haematobium is found primarily in the venous plexus of the bladder. It is within these blood vessels that paired adult worms produce eggs, which are then deposited in the surrounding tissues (2, 19). Egg production usually begins several weeks after infection, once sexual maturity and pairing have occurred. These eggs play a central role in the pathogenesis of schistosomiasis, as their traversal through host tissues triggers intense immune responses and granuloma formation, leading to much of the disease’s morbidity (25).

To ensure transmission, the eggs must actively traverse host tissues to reach the intestinal or vesical lumen (127). This process is mediated by egg-secreted enzymes such as metalloproteases, which degrade the extracellular matrix and facilitate tissue penetration. Inhibition of these proteases has been shown to impair tissue digestion and significantly reduce egg excretion (128), highlighting their essential role in parasite transmission. However, many eggs fail to reach the lumen and become lodged in host tissues (26), where they elicit potent immune responses.

Mature eggs adhere to the endothelium and rapidly recruit immune cells, particularly eosinophils, macrophages, CD4^+^ T helper cells and fibroblasts, culminating in granuloma formation, a protective yet pathological structure that forms around trapped eggs. These granulomas serve a dual purpose: they encapsulate toxic egg antigens to limit local tissue damage, and they facilitate egg extrusion into the lumen. A compromised host immune response also results in a reduced rate of egg excretion, supporting the hypothesis that the granulomatous reaction is not only a consequence of infection but also facilitates the translocation of eggs across host tissues (129).

Despite this protective intent, chronic granulomatous inflammation leads to progressive fibrosis and organ dysfunction. Interestingly, only mature eggs induce granulomas, whereas immature eggs fail to recruit macrophages, suggesting the eggshell is initially immunologically inert. This likely provides a temporal window for the miracidium to mature before triggering host immunity (130).

Several components of Schistosoma eggs possess immunomodulatory activity and contribute to the distinctive immunological milieu associated with infection. These include both molecules that are actively excreted or secreted by the eggs, and those identified through the analysis of egg homogenates, such as many components of the soluble egg antigens (SEA). There is a broad consensus that Schistosoma eggs orchestrate a complex cytokine network that promotes Th2 polarization, facilitates granuloma formation, and tempers excessive inflammation.

Eggs trapped in liver capillaries activate endothelial cells and hepatic stellate cells (HSC) to secrete chemokines, CCL2 in particular, to recruit circulating leukocytes. Macrophages are a major component of Schistosoma-induced granuloma. Cytokine microenvironment, dominated by the Th2 cytokines IL-4 and IL-13, promotes the macrophage polarization toward a M2-like phenotype expressing arginase 1 and Fizz-I. M2 polarization is also influenced by direct contact with the eggs themselves. Zhu et al. demonstrated that peritoneal macrophages isolated from healthy mice exhibited elevated expression of chemokines such as CCL2, CCL17, and CCL22, along with IL-10 and Arg-1, following stimulation with S. japonicum SEA (131). Similarly, Xu et al. reported an increased production of IL-10 in RAW264.7 macrophages upon exposure to S. japonicum SEA (132).

The Th1-Th2 shift following egg deposition, with a progressive reduction in proinflammatory cytokines such as IFN-γ and TNF-α, represents a crucial step in the immune response to the parasite. This is demonstrated by the high mortality rate in mice deficient in IL-4 and IL-4/IL-10 (133–135). Conversely, neutralization of IFN-g or IL-12 results in accentuated fibrosis (136).

Time course experiments in murine model indicate a peak of Th2 cytokines (IL-4, IL-5 and IL-13) soon after egg deposition in the liver and an increase of IL-10 at later time point (137), revealing the onset of regulatory mechanisms that dampen the immune response. Overall, SEA has a dual effect on the immune system. On one side it promotes the development of Th2 cells, on the other, it induces regulatory mechanisms mediated by direct, such as induction of T cell apoptosis via FAS-FASL mediated pathway and the upregulation of PD-1 of T cells (138) and indirect effects, via modulation of the dendritic cell activation. In particular, DC exposed to SEA show impaired maturation following TLR stimulation, characterized by reduced expression of MHC class II molecules and other maturation markers (139).

Egg-derived glycolipids selectively stimulate monocytes to produce IL-10, IL-6, and TNF-α, underscoring the unique immunoregulatory properties of egg components (140) Among these, IPSE/α-1—a major glycoprotein released from the subshell region—induces IL-4 production and strong antibody responses, initiating Th2 immunity (141). SEA-derived Omega-1 potently drives Th2 polarization by acting on human dendritic cells via mannose receptor–mediated uptake, while it promotes a Th2/tolerogenic environment in DC by upregulating OX40L, reducing TNF-α production (142, 143) and inducing PGE2 synthesis via ERK signaling through Dectin-1 and Dectin-2 (144). IL-33 contributes to the early phase of type 2 responses, particularly in the gut, though it appears dispensable for parasite maturation and egg deposition (145). SEA also promote IL-4–producing cells, including eosinophils and mononuclear cells, which support polyclonal B cell activation (146). SEA influences B cell function by inducing IL-10 and CD86 expression in marginal zone B cells, independently of macrophages, and promotes regulatory B cell (Breg) differentiation via components like IPSE/α-1, but not omega-1 or kappa-5 (147). Egg-derived extracellular vesicles enriched in Sja-miR-71a suppress neutrophil and macrophage extracellular trap formation through the Sema4D/PPAR-γ/IL-10 axis, further reinforcing SEA’s anti-inflammatory profile (148). Beyond Th2 responses, IL-17–producing cells contribute to hepatic granuloma formation and fibrosis, correlating with ICOS expression and implicating Th17 cells in pathological remodeling (149). Conversely, IL-22 appears protective: schistosome eggs upregulate IL-22 while suppressing its binding protein, and IL-22-producing T cells attenuate IL-13-driven M2 macrophage polarization and fibrogenesis, reducing collagen synthesis and hepatic stellate cell proliferation (150, 151).

Consequence of the release of Th2 cytokine in the granuloma is the activation of hepatic stellate cells to produce collagen thus inducing periportal fibrosis (152). Both IL-4 and IL-13 are involved in the HSC activation. Nevertheless, IL-4 blockade failed to prevent the fibrotic process whereas IL-13 is now considered the major driving cytokine driving collagen production by HSC (153, 154) since blockade of the IL-4Ra, common receptor for IL-4 and IL-13, or directly IL-13 is highly effective in reducing liver fibrosis due to schistosoma egg deposition and increase survival (155, 156). As infections progress into chronicity, Type 2 responses decline, and regulatory responses prevail (5, 2931). Down-modulation of Type 2 responses and suppression of severe disease is thought to be primarily mediated by IL-10 (32), with its secretion attributed to Regulatory B cells (Bregs) (33, 34) and T cells (Tregs) (30, 35–37). Additionally, SEA promote apoptosis of CD4^+^ T cells via FasL-mediated pathways (138). In parallel, the upregulation of PD-1 on CD4^+^ T cells serves as an immune checkpoint that restrains pathogenic Th2 responses; its blockade exacerbates hepatic immunopathology without affecting egg burden (138). Regulatory T cells (CD4^+^CD25^+^FoxP3^+^) further suppress inflammation and limit tissue fibrosis during chronic infection, particularly within the colonic granulomas (157).

## Conclusions and future directions

**Figure 2:**
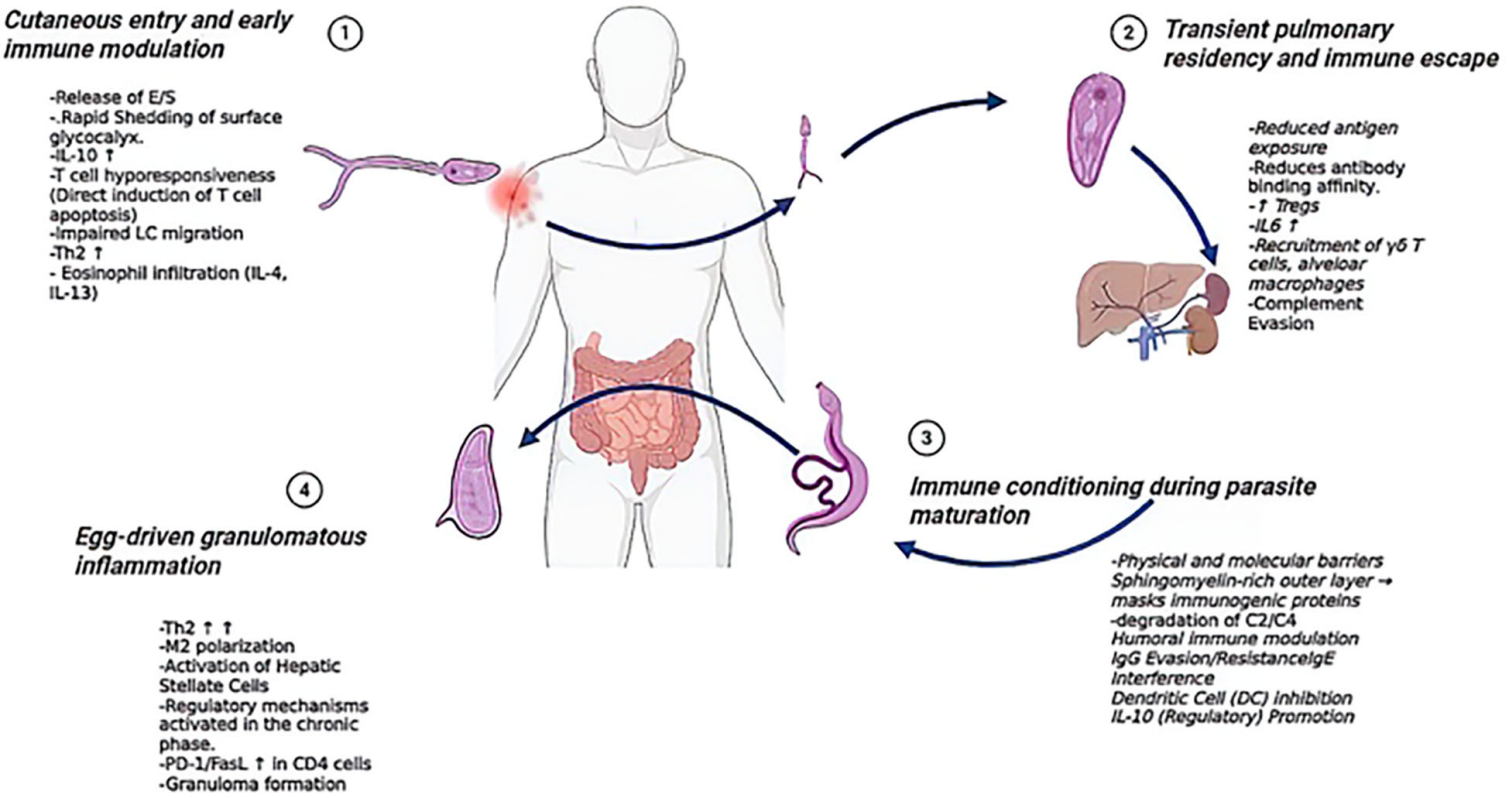
Stage-specific immune modulation during the *Schistosoma* life cycle. Overview of host immune modulation at distinct stages of *Schistosoma* infection (1). Skin penetration is associated with the release of excretory/secretory (E/S) products, glycocalyx shedding, IL-10–mediated T cell hyporesponsiveness, impaired Langerhans cell (LC) migration, and eosinophil infiltration (2). During transient lung residency, schistosomula reduce antigen exposure, evade antibody and complement responses, and promote regulatory T cells (Tregs) and IL-6 production (3). Parasite maturation involves the establishment of molecular barriers, inhibition of dendritic cells (DC), degradation of complement components, interference with IgG-mediated immunity, and promotion of regulatory IL-10 responses (4). Egg deposition induces Th2-driven granulomatous inflammation, M2 macrophage polarization, activation of hepatic stellate cells, and regulatory mechanisms involving PD-1/FasL expression in CD4+ T cells. DC, dendritic cell; E/S, excretory/secretory products; LC, Langerhans cell; Treg, regulatory T cell.

Schistosomiasis represents a paradigmatic case of host–parasite interaction in which survival depends on the capacity of the parasite to modulate and exploit host immunity (**Figure 2**). Notably, schistosomes employ a wide repertoire of immunomodulatory and immune-evasive strategies that enable them to complete their life cycle and disseminate within the host. Moreover, these mechanisms are not restricted to local sites of infection; rather, they exert systemic effects that reshape immune homeostasis at the organismal level. In this context, the parasite not only attenuates inflammatory responses to facilitate its persistence, but also leverages host immunity to promote egg expulsion, thereby ensuring transmission to the external environment. Importantly, the finely tuned immunoregulatory activities of Schistosoma extend beyond parasite survival, influencing the trajectory of autoimmune and inflammatory diseases in infected individuals, which often diverge from their classical pathophysiological course. Recent research has increasingly focused on exploiting the immunomodulatory properties of schistosome-derived molecules, particularly their capacity to promote Th2 responses, as a therapeutic approach for human disease (158). This strategy is being investigated in a variety of pathological contexts where excessive inflammation or autoimmunity plays a central role. For instance, experimental models have shown that SEA or other schistosoma derivates can ameliorate conditions such as inflammatory bowel disease (159, 160), multiple sclerosis (161), rheumatoid arthritis (162–164), diabetes (165, 166) and asthma (167, 168) and many other conditions. Many reviews have comprehensively summarized these advances, highlighting how parasite-derived immunomodulation is being translated into potential interventions aimed at restoring immune balance and reducing tissue damage in chronic inflammatory and autoimmune disorders (21, 24, 169).

An unavoidable consequence of the immunomodulatory capacity of Schistosoma is the persistent difficulty in developing an effective vaccine. Despite decades of research aimed at identifying antigens capable of blocking cercarial penetration or limiting parasite dissemination, results have remained disappointing. The parasite’s ability to manipulate host immunity prevents the establishment of durable protective responses, thereby undermining conventional vaccine strategies. Research efforts have concentrated on numerous vaccine candidates that remain under investigation at the preclinical stage (170). Among these, only four candidates—based on Schistosoma proteins or recombinant proteins such as Sm14 (171–173), Sm−p80 (174) (173), rSh28GST (175) (174) and Sm−TSP−2 (176, 177) have advanced to clinical evaluation (**Table 1**), although only one clinical trial has published its results to date (178). Despite research efforts, current efficacy outcomes remain insufficient. Although some candidates have shown encouraging pre−clinical performance, most still fail to reach the protective levels required for an effective schistosomiasis vaccine (179–183).

**Table 1:** Overview of clinical trials (completed, active, or of unknown status) of schistosomiasis vaccine candidates.

ClinicalTrials.gov ID	Antigen/target protein	Trial phase	Participant age group	Status
NCT05999825	Sm-p80	2	Adult	Unknown status
NCT01512277	rSh28GST	1	Adult	Completed
NCT01154049	Sm14	1	Adult	Completed
NCT03110757	Sm-TSP-2	1	Adult	Completed
NCT05292391	Sm-p80	1	Adult	Completed
NCT05762393	Sm-p80	1	Adult	Active
NCT03041766	Sm14	2	Adult	Completed
NCT05658614	Sm14	2	Adult	Unknown status
NCT02337855	Sm-TSP-2	1	Adult	Completed
NCT03910972	Sm-TSP-2	1, 2	Adult	Completed
NCT00870649	rSh28GST	3	Child	Completed
NCT03799510	Sm14	3	Child	Completed

This reality highlights the need for innovative strategies that integrate immunology and host–parasite interaction studies to advance therapy development against schistosomiasis. For these reasons, it becomes increasingly important to deepen our understanding of how Schistosoma manipulates host immune responses. Clarifying the mechanisms underlying parasite-driven immunoregulation is a necessary step toward the development of therapies capable of overcoming the barriers that have thus far limited progress in schistosomiasis vaccine research. At the same time, a better understanding of schistosome immune biology may provide opportunities to evaluate parasite-derived molecules as potential therapeutic agents. In this regard, continued investigation of schistosome–host interactions is important not only for advancing strategies to control schistosomiasis, but also for assessing their possible relevance in the development of novel immunomodulatory approaches applicable to a wider range of human diseases.
